# A Novel Risk-Adjusted Metric to Compare Hospitals on Their Antibiotic Prescribing at Hospital Discharge

**DOI:** 10.1093/cid/ciae224

**Published:** 2024-04-24

**Authors:** Daniel J Livorsi, James A Merchant, Hyunkeun Cho, Matthew Bidwell Goetz, Bruce Alexander, Brice Beck, Michihiko Goto

**Affiliations:** Center for Access and Delivery Research and Evaluation, Iowa City Veterans Affairs Health Care System, Iowa City, Iowa, USA; Division of Infectious Diseases, University of Iowa Carver College of Medicine, Iowa City, Iowa, USA; Department of Biostatistics, University of Iowa, Iowa City, Iowa, USA; Department of Biostatistics, University of Iowa, Iowa City, Iowa, USA; VA Greater Los Angeles Healthcare System, Los Angeles, California, USA; David Geffen School of Medicine at the University of California, Los Angeles, California, USA; Center for Access and Delivery Research and Evaluation, Iowa City Veterans Affairs Health Care System, Iowa City, Iowa, USA; Center for Access and Delivery Research and Evaluation, Iowa City Veterans Affairs Health Care System, Iowa City, Iowa, USA; Center for Access and Delivery Research and Evaluation, Iowa City Veterans Affairs Health Care System, Iowa City, Iowa, USA; Division of Infectious Diseases, University of Iowa Carver College of Medicine, Iowa City, Iowa, USA

**Keywords:** antibiotic stewardship, hospital discharge, metrics, risk-adjustment, pneumonia, community-acquired

## Abstract

**Background:**

Antibiotic overuse at hospital discharge is common, but there is no metric to evaluate hospital performance at this transition of care. We built a risk-adjusted metric for comparing hospitals on their overall post-discharge antibiotic use.

**Methods:**

This was a retrospective study across all acute-care admissions within the Veterans Health Administration during 2018–2021. For patients discharged to home, we collected data on antibiotics and relevant covariates. We built a zero-inflated, negative, binomial mixed model with 2 random intercepts for each hospital to predict post-discharge antibiotic exposure and length of therapy (LOT). Data were split into training and testing sets to evaluate model performance using absolute error. Hospital performance was determined by the predicted random intercepts.

**Results:**

1 804 300 patient-admissions across 129 hospitals were included. Antibiotics were prescribed to 41.5% while hospitalized and 19.5% at discharge. Median LOT among those prescribed post-discharge antibiotics was 7 (IQR, 4–10) days. The predictive model detected post-discharge antibiotic use with fidelity, including accurate identification of any exposure (area under the precision-recall curve = 0.97) and reliable prediction of post-discharge LOT (mean absolute error = 1.48). Based on this model, 39 (30.2%) hospitals prescribed antibiotics less often than expected at discharge and used shorter LOT than expected. Twenty-eight (21.7%) hospitals prescribed antibiotics more often at discharge and used longer LOT.

**Conclusions:**

A model using electronically available data was able to predict antibiotic use prescribed at hospital discharge and showed that some hospitals were more successful in reducing antibiotic overuse at this transition of care. This metric may help hospitals identify opportunities for improved antibiotic stewardship at discharge.

## Teaser

We developed a model using electronically available data to predict antibiotics prescribed at hospital discharge. A risk-adjusted metric based on this model showed that some hospitals are more successful in reducing antibiotic overuse at this transition of care.

Antibiotic resistance is a persistent and growing public health problem. More judicious use of antibiotics will slow the emergence and spread of antibiotic resistance [1]. Therefore, efforts to improve the measurement and prescription of appropriate antibiotics are needed.

Early efforts at antibiotic stewardship have largely focused on antibiotic prescribing that takes place within hospitals. Based on a 2017 Cochrane review, there is “high-certainty evidence” that core antibiotic stewardship strategies improve guideline-concordant antibiotic prescribing and reduce the duration of inpatient antibiotic therapy [2]. However, approximately 40% of all hospital-related antibiotic exposure is prescribed at the point of hospital discharge [3, 4]. Studies have found that 40–79% of post-discharge antibiotic use is either unnecessary or suboptimal, and the most common reason for this assessment is the excessive duration of therapy [5–8].

Standard metrics for hospital-based antibiotic stewardship programs do not capture post-discharge antibiotics, and accurate measurement of post-discharge antibiotic use is difficult [9]. For one, inpatient and outpatient health data are often not integrated. In addition, recommended treatment courses for the patient to take after discharge are sometimes dispensed outside the system that provided the patient's hospital care.

The Veterans Health Administration (VHA), the largest integrated healthcare system in the United States, provides a unique opportunity to evaluate post-discharge antibiotic use because these prescriptions are entirely dispensed from within the VHA system. In this study, we sought to leverage this data source to develop a risk-adjusted, hospital-level metric on post-discharge antibiotic use.

## METHODS

We performed a retrospective study across all acute-care admissions within the VHA during 2018–2021 and constructed a predictive model for post-discharge antibiotic therapy.

The Institutional Review Board (IRB) of the University of Iowa and Iowa City Veterans Health Care System approved this study. Waiver for informed consent was granted by the IRB for this retrospective cohort.

### Data Sources

We accessed national administration data from the VHA Corporate Data Warehouse (CDW) via the VHA Informatics and Computing Infrastructure. This included data on patient demographics, comorbidities, procedures, primary services, and discharge diagnoses of infection. Antibiotic use and immunosuppressive medications were identified in the Barcode Medication Administration (BCMA) pharmacy data domain of the CDW and from outpatient medication files (Supplementary Table 1).

### Patients and Hospitals

We included patient-admissions if they were admitted during 2018–2021 to an acute-care bed in any VHA medical center, including an intensive care unit, step-down unit, medical or surgical unit, or observational bed, and were discharged to the community (ie, home or self-care with or without home health services). Patient-admissions were excluded if their discharge status was classified as either (1) death or (2) transfer to another acute-care or post–acute-care facility. These transfers were excluded because capture of post-discharge antibiotic use would be incomplete.

### Variables

For each patient-admission, we collected data on all antibacterials (hereafter “antibiotics”) included in the National Healthcare Safety Network’s (NHSN's) Antimicrobial Use and Resistance Protocol [10]. This included inpatient antibiotics administered via the following routes: intravenous, intramuscular, digestive tract (eg, oral), or respiratory tract. Post-discharge antibiotics were defined as oral antibiotics dispensed from the outpatient pharmacy during the discharge period [3]. We assumed that all outpatient oral antibiotics dispensed during this time frame were initiated by the patient on the day following discharge and were taken for a duration equal to the days’ supply of the dispensed prescription. If more than 1 antibiotic was prescribed at discharge, we assumed all agents were taken concurrently.

Using a modified version of the Elixhauser comorbidity index, we identified patients as having comorbidities based on International Classification of Diseases, Tenth Revision (ICD-10), codes from outpatient and inpatient encounters over the 12 months prior to the index visit and from the index admission itself [11]. We specifically captured comorbidities previously defined as being causally (n = 14) or indeterminately (n = 6) related to appropriate antibiotic use [12]. Diagnoses of bacterial infection were based on ICD-10 codes applied at hospital discharge, as defined by a publicly available Agency for Healthcare Research and Quality resource [13].

### Outcomes

The primary outcome was post-discharge antibiotic use, which was further quantified as length of therapy (LOT). Post-discharge LOT equals the number of days of antibiotic exposure prescribed at discharge, regardless of the number of agents prescribed per day. Outliers for post-discharge LOT were defined by 5 times the interquartile range (IQR) of LOT in all patients who received any antibiotics, which equated to an LOT equal to 30 days [14]. These outliers were excluded from the model, in part, because these prescriptions may reflect chronic antibiotic prophylaxis or suppression. In addition, because hospitals differ in their use of oral versus parenteral antibiotics for osteoarticular and other complicated infections, this exclusion was important to not unfairly penalize hospitals that are more frequent users of oral therapy [15].

### Statistical Analysis

We conducted an analysis at the level of patient-admissions using zero-inflated negative binomial mixed models with 2 random intercepts for each hospital modeled jointly following a bivariate normal distribution. A zero-inflated approach was used because (1) there was a high proportion of patients with no post-discharge antibiotic exposure and (2) there were patients who, given the nature of their illness, likely had zero probability of being prescribed post-discharge antibiotics. This approach allowed us to model whether a patient-admission received any post-discharge antibiotics (zero-inflated component) and, if so, how much (count component) [16]. By including both these components, we accounted for the reality that the association between covariates and the likelihood of receiving any post-discharge antibiotics may be different than the association between covariates and the duration of post-discharge antibiotics.

We initially divided the data into 2 periods (2018–2019 and 2020–2021) to account for potential differences in antibiotic prescribing during the coronavirus disease 2019 (COVID-19) pandemic. However, coefficients from these 2 models were found to be very similar (Supplementary Table 2), so we decided to analyze all 4 years together.

Data for 2018–2021 were split 1:1 into training and testing sets to evaluate model performance by using mean absolute error. Covariates in this model were factors postulated to influence post-discharge antibiotic use and duration: patient age, sex, medical versus surgical specialty at time of discharge, direct transfer to VHA from another hospital, body mass index, receipt of immunosuppressive medications, selected Elixhauser comorbidities, immunodeficiency, discharge diagnoses of bacterial infection, and the number of days of inpatient antibiotic exposure, which was treated as an ordinal variable (0 days, 1–2 days, 3–7 days, >7 days) because the relationship between the duration of inpatient antibiotics and the primary outcome did not change in a linear fashion.

We used the area under the precision-recall curve to assess classification accuracy for the zero-inflated portion of the model. A higher area under the precision-recall curve indicated that the model was better able to identify patients who received any post-discharge antibiotics compared with random chance guessing. We used mean absolute error to evaluate the model's accuracy in predicting LOT. We did not use a process for variable selection, because our aim was to predict antibiotic use and a model with all variables was successfully fitted to the data with a large sample.

### Assessing Hospital Performance With This Risk-Adjusted Method

The performance of hospitals was determined by 2 random intercepts, which captured the variance between hospitals. Exponentiation of these random intercept values calculated the conditional odds ratio for any antibiotic prescribing at discharge (the zero-inflated component) and the rate ratio for post-discharge antibiotic LOT (the count component) at a specific hospital. For both components, a hospital with a negative predicted random intercept had lower observed post-discharge antibiotic use than would be expected in a hospital with identical fixed effects (eg, the same patient characteristics) but a random intercept set to zero. The expected frequency or duration of post-discharge antibiotics indicates how a hospital would have prescribed if, given its fixed effects, it had performed in the same way as the reference population.

Hospitals that had negative random intercepts for both the zero-inflated and count components of the model were categorized as group 1; after adjusting for fixed effects, these hospitals prescribed antibiotics less frequently at discharge and used shorter courses when post-discharge antibiotics were prescribed. Hospitals that had positive random intercepts for both components were categorized as group 4; these hospitals prescribed antibiotics more frequently at discharge and used longer courses when post-discharge antibiotics were prescribed. Group 2 hospitals prescribed antibiotics more frequently at discharge (positive random intercept for the zero-inflated component) but prescribed shorter courses (negative random intercept for the count component). Group 3 hospitals prescribed antibiotics less frequently at discharge (negative random intercept for the zero-inflated component) but prescribed longer courses (positive random intercept for the count component).

### Comparing Hospital Performance on the Risk-Adjusted Metric to Antibiotic Prescribing for Community-Acquired Pneumonia

We compared hospital performance on this risk-adjusted metric (groups 1–4) with a more intuitive assessment of antibiotic-prescribing quality: total antibiotic duration (inpatient + post-discharge) for uncomplicated cases of community-acquired pneumonia (CAP). Eligible cases of CAP were identified using the criteria shown in Supplementary Tables 3 and 4, which included the following: a discharge diagnosis of pneumonia; chest imaging within the 48 hours before/after admission; antibiotics started less than 48 hours after admission; receipt of inpatient with or without post-discharge antibiotics for LOT ≥4; the absence of a lung abscess, an empyema, and other infection types; no pleural drainage procedures; and no immunosuppression.

For groups 1–4, we calculated (a) mean total antibiotic duration for CAP and (b) the percentage of patients with CAP who received the shortest effective antibiotic duration. An LOT of 6 or fewer days was chosen as the upper cutoff for “b” because patients receiving an antibiotic dosed more frequently than every 24 hours could have received antibiotics on 6 unique calendar days, even though the total duration was equal to 120 hours, or 5 days. Professional guidelines on CAP acknowledge that a total antibiotic duration of 5 days is appropriate for most patients [17]. We compared “b” across patients at hospitals within each group (1–4) by using a generalized linear mixed model with the logit link function. This model included indicator variables for the type of random intercept group and a random intercept for each hospital.

## RESULTS

**Figure 1.**
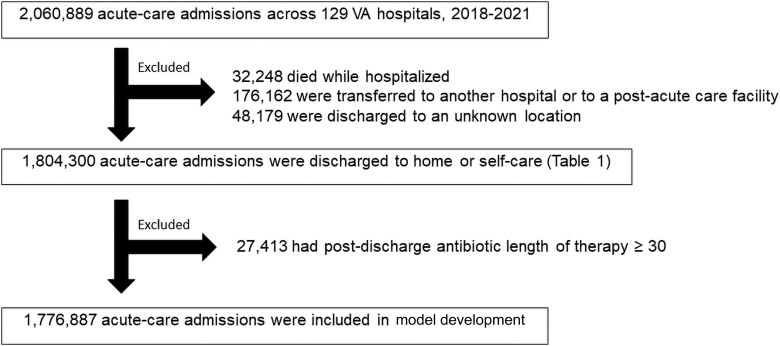
Flowchart for how the study cohort was constructed. Abbreviation: VA, Veterans Affairs.

There were 2 060 889 admissions across 129 hospitals during 2018–2021, among whom 32 248 (1.6%) died, 176 162 (8.5%) were transferred to another hospital or to a post–acute-care facility, and 48 179 (2.3%) were discharged to an unknown location (Figure 1). Baseline characteristics of the remaining 1 804 300 patient-admissions are shown in Table 1.

**Table 1.** Characteristics of Patients Who Were Discharged Home or to Self-Care After an Acute-Care Admission (2018–2021), Stratified by Whether Oral Antibiotics Were Prescribed at Hospital Discharge

	Total (n = 1 804 300)	No Post-discharge Antibiotics (n = 1 452 944)	Post-discharge Antibiotics Prescribed (n = 351 356)
Age, mean (SD), y	67.8 (12.9)	67.6 (13.0)	68.4 (12.6)
Male sex	1 691 350 (93.7)	1 360 772 (93.7)	330 578 (94.1)
Body mass index			
Normal	424 501 (23.5)	340 702 (23.4)	83 799 (23.9)
Overweight/obese	1 200 086 (66.5)	966 697 (66.5)	233 389 (66.4)
Missing	140 216 (7.8)	114 774 (7.9)	25 442 (7.2)
Underweight	39 497 (2.2)	30 771 (2.1)	8726 (2.5)
Transferred from another hospital	39 528 (2.2)	31 975 (2.2)	7553 (2.2)
Medical specialty at discharge	1 449 842 (80.4)	1 163 849 (80.1)	285 993 (81.4)
Comorbidities^a^			
Congestive heart failure	558 994 (31.0)	459 592 (31.6)	99 402 (28.3)
Chronic lung disease	683 868 (37.9)	529 358 (36.4)	154 510 (44.0)
Diabetes mellitus	810 260 (44.9)	648 901 (44.7)	161 359 (45.9)
Immunodeficiency^b^	93 262 (5.2)	70 640 (4.9)	22 622 (6.4)
Liver disease	315 571 (17.5)	257 150 (17.7)	58 446 (16.6)
Metastatic cancer	107 595 (6.0)	85 973 (5.9)	21 622 (6.2)
Neurological disorder^c^	389 646 (21.6)	322 101 (22.2)	67 545 (19.2)
Peptic ulcer disease	65 865 (3.7)	54 837 (3.8)	11 028 (3.1)
Pulmonary circulation disorders	92 854 (5.2)	75 790 (5.2)	17 064 (4.9)
Peripheral vascular disease	412 636 (22.9)	330 182 (22.7)	82 454 (23.5)
Renal disease or dialysis	505 966 (28.0)	410 546 (28.3)	95 420 (27.2)
Rheumatic disorder	51 733 (2.9)	40 624 (2.8)	11 109 (3.2)
Substance abuse^d^	481 891 (26.7)	399 902 (27.5)	81 989 (23.3)
Valvular disease	232 256 (12.9)	194 349 (13.4)	37 907 (10.8)
Weight loss	250 334 (13.9)	200 761 (13.8)	49 573 (14.1)
Immunosuppressive medications^e^	95 694 (5.3)	73 643 (5.1)	22 051 (6.3)
Discharge diagnoses			
COPD exacerbation	119 921 (6.7)	66 908 (4.6)	53 013 (15.1)
Intra-abdominal and biliary infections	68 561 (3.8)	38 467 (2.7)	30 094 (8.6)
Miscellaneous bacterial infection^f^	280 780 (15.6)	147 864 (10.2)	132 916 (37.8)
Osteoarticular infections	52 732 (2.9)	34 440 (2.4)	18 292 (5.2)
Pneumonia	133 943 (7.4)	64 925 (4.5)	69 018 (19.6)
Skin and soft tissue infection	152 690 (8.5)	80 963 (5.6)	71 727 (20.4)
Urinary tract infection	131 419 (7.3)	67 657 (4.7)	63 762 (18.2)
Inpatient antibiotic exposure			
None	1 054 992 (58.5)	1 032 182 (71.0)	22 810 (6.5)
1–2 d	313 813 (17.4)	196 965 (13.6)	116 848 (33.3)
3–7 d	335 228 (18.6)	155 024 (10.7)	180 204 (51.3)
>7 d	100 267 (5.6)	68 773 (4.7)	31 494 (9.0)

Data are presented as n (%) unless otherwise indicated.

Abbreviations: COPD, chronic obstructive pulmonary disease; SD, standard deviation.

^a^These comorbidities were selected because a prior Delphi panel determined that they were causally or indeterminately related to appropriate inpatient antibiotic use [12]. Note that some conditions identified by the Delphi panel have been combined together.

^b^Immunodeficiency includes diagnoses of leukemia, lymphoma, human immunodeficiency virus (HIV) infection, bone marrow transplantation, or solid-organ transplantation.

^c^Neurological disorders include paralysis and other neurological disorders—eg, Parkinson's disease, multiple sclerosis, epilepsy, etc.

^d^Substance abuse includes alcohol and/or drug abuse.

^e^Immunosuppressive medications included receipt of chemotherapeutic agents within 30 d before the admission or during the admission itself or receipt of immune-modulating medications within 90 d before the admission or during the admission itself (Supplementary Table 1).

^f^Miscellaneous bacterial infections included endocarditis, central nervous system infections, complicated pneumonia (eg, empyema, lung abscess), sepsis, and any other bacterial infections that were not otherwise categorized.

There were 749 308 (41.5%) admissions exposed to inpatient antibiotics and 351 356 (19.5%) exposed to post-discharge antibiotics. The median LOT for inpatient antibiotics was 3 (IQR, 2–5) days. The median post-discharge LOT in those who were exposed was 7 (IQR 4–10) days. There were 27 413 (1.5%) patient-admissions with a post-discharge LOT of 30 days or more. Characteristics of patients with an LOT of 30 or more are shown in Supplementary Table 5. Notable differences between patients with an LOT of 30 or more versus an LOT of 1–29 days were the prevalence of immunodeficiency (18.4% vs 5.4%), use of immunosuppressive medications (17.0% vs 5.4%), miscellaneous bacterial infections (48.0% vs 37.0%), and osteoarticular infections (20.7% vs 3.9%).

### Predictive Model for Post-discharge Length of Therapy

Supplementary Table 6 shows the parameters for the predictive LOT model, which excluded outliers (ie, LOT ≥30 days). The zero-inflated component detected post-discharge antibiotic use with fidelity, including accurate identification of admissions with any post-discharge antibiotic exposure (area under the precision-recall curve = 0.97). The model reliably predicted the number of days of post-discharge LOT in those who were exposed (mean absolute error = 1.48) (Supplementary Figure 1).

### Hospital Performance on the Novel Risk-Adjusted Metric

**Figure 2.**
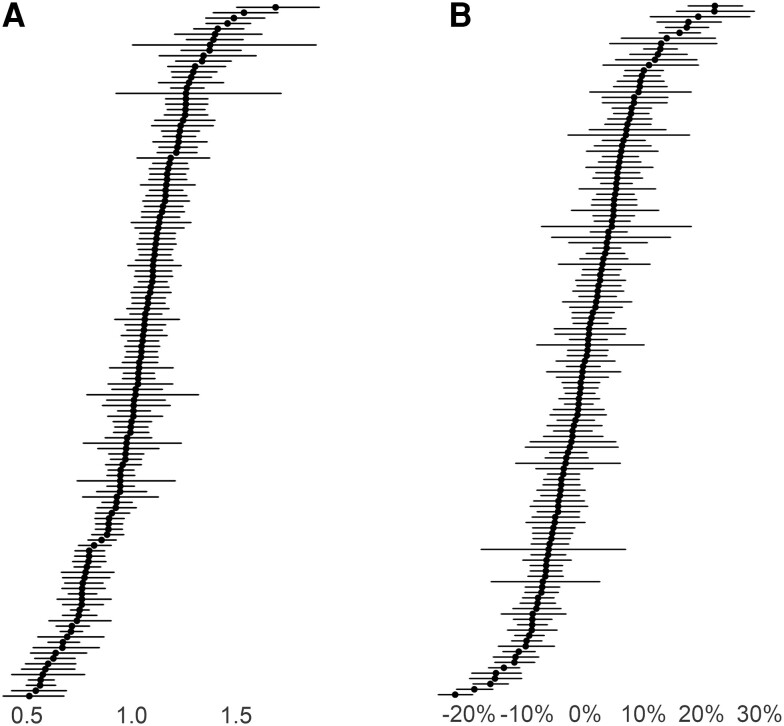
*A*, Caterpillar plot of the conditional odds ratio for any antibiotic prescribing at discharge for 129 hospitals. The points on the horizontal axis represent the conditional odds ratio at each hospital for any antibiotic prescribing at discharge compared with a hospital with the expected frequency of antibiotic prescribing at discharge. The error bars represent a 95% confidence interval (CI) around that predicted value for each hospital. Hospitals with CIs that are entirely above or below 1 can be interpreted as having significantly more frequent or less frequent antibiotic prescribing, respectively, holding all fixed effects equal. *B*, Caterpillar plot of the rate ratio for post-discharge length of antibiotic therapy at 129 hospitals. The points on the horizontal axis represent the relative percentage difference in post-discharge antibiotic length of therapy (LOT) between each hospital and a hospital that prescribes the expected post-discharge LOT. The error bars represent the 95% CI around that predicted value for each hospital. Hospitals with CIs that are entirely above or below zero can be interpreted as having significantly longer or shorter LOT, respectively, holding all fixed effects equal.

**Figure 3.**
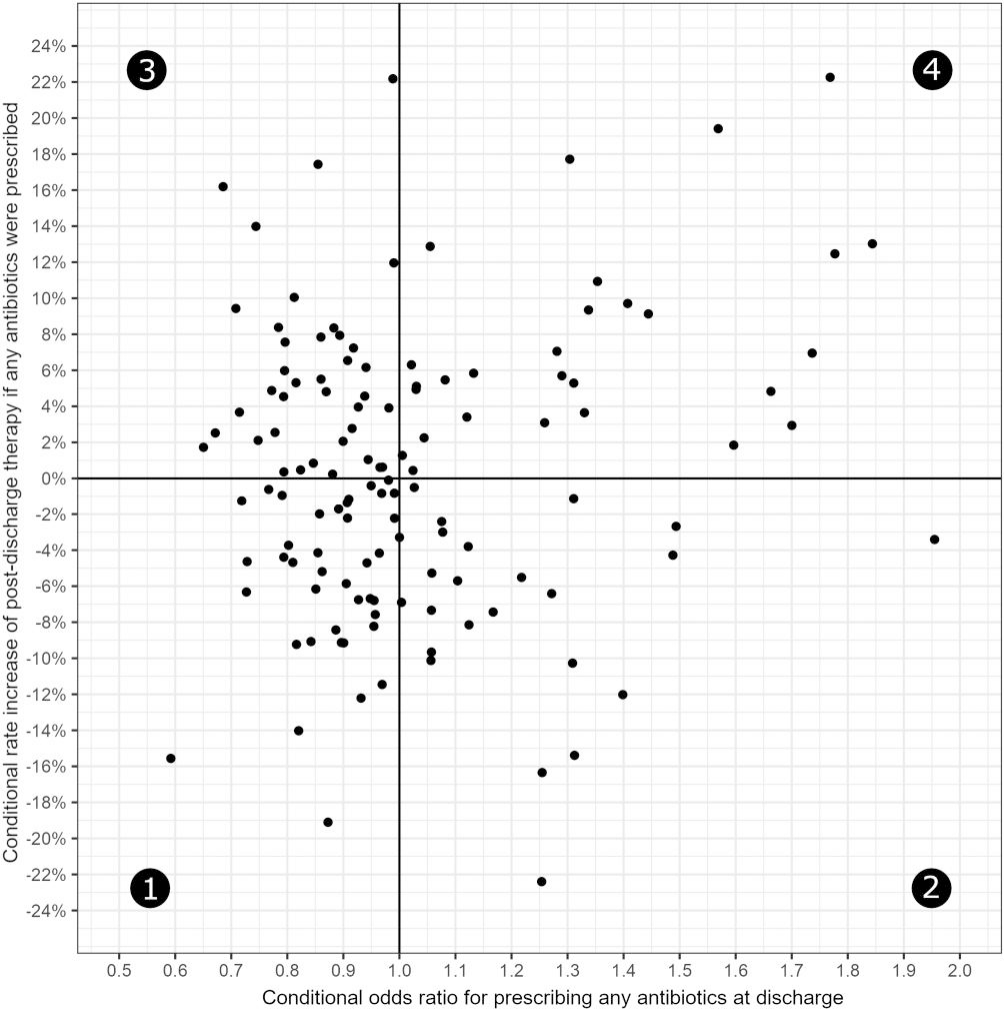
Risk-adjusted comparison of post-discharge antibiotic-prescribing frequency and duration across 129 hospitals. Group 1 hospitals had less frequent post-discharge antibiotic prescribing and used shorter post-discharge antibiotic duration. Group 2 hospitals had more frequent post-discharge antibiotic prescribing and used shorter post-discharge antibiotic durations. Group 3 had less frequent post-discharge antibiotic prescribing and used longer post-discharge antibiotic durations. Group 4 hospitals had more frequent post-discharge antibiotic prescribing and used longer post-discharge antibiotic durations.

Figure 2 shows how each hospital performed in the zero-inflated and count components, respectively. Figure 3 shows how hospitals performed when both model components were considered concomitantly (ie, the risk-adjusted metric). There were 39 (30.2%) hospitals in group 1, 24 (18.6%) in group 2, 38 (29.5%) in group 3, and 28 (21.7%) in group 4.

### Comparison Between the Risk-Adjusted Metric and Antibiotic Duration for Community-Acquired Pneumonia

There were 41 076 cases of uncomplicated CAP, which accounted for 5.5% of all inpatients exposed to antibiotics. The median number of CAP cases per hospital was 286 (IQR, 181–407); all but 4 (3.1%) hospitals had 50 or more cases. The median inpatient antibiotic duration was 4 (IQR, 2–5) days and the median post-discharge duration was 3 (IQR, 0–5) days. In all, 70.3% of patients with CAP received post-discharge antibiotics. Table 2 shows how antibiotic duration differed across groups 1–4. Compared with patients at group 4 hospitals, patients were more likely to receive the shortest effective antibiotic duration for CAP at group 1 hospitals (odds ratio [OR], 2.97; 95% confidence interval [CI], 2.27–3.90; *P* < .001), group 2 hospitals (OR, 2.52; 95% CI, 1.86–3.43; *P* < .001), and group 3 hospitals (OR, 2.18; 95% CI, 1.66–2.86; *P* < .001).

**Table 2.** Comparison of Total Antibiotic Duration for Uncomplicated Community-Acquired Pneumonia, Stratified by Hospital Performance on the Risk-Adjusted Metric

Group	Frequency of Antibiotic Prescribing at Discharge^a^	Post-discharge Antibiotic Duration^b^	No. of Patients	Mean Antibiotic Duration, d	% of Patients Prescribed the Shortest Effective Antibiotic Duration	Odds Ratio for Shortest Effective Duration (95% CI)
1	Less often than expected	Shorter than expected	13 061	7.3	49.3	2.97 (2.27–3.90)
2	More often than expected	Shorter than expected	6616	7.4	48.5	2.52 (1.86–3.43)
3	Less often than expected	Longer than expected	13 230	7.9	42.4	2.18 (1.66–2.86)
4	More often than expected	Longer than expected	8169	8.6	29.0	Reference

Abbreviation: CI, confidence interval.

^a^This column corresponds to the hospital's random intercept in the zero-inflated component of the model for overall post-discharge antibiotic use.

^b^This column corresponds to the hospital's random intercept in the count component of the model for overall post-discharge antibiotic use.

## DISCUSSION

A model using electronically available data was able to predict post-discharge antibiotic use, including any antibiotic exposure and the length of antibiotic therapy, given at hospital discharge. The model, which adjusted for relevant covariates, showed variation across hospitals in their overall post-discharge antibiotic use. One of 5 hospitals (group 4) prescribed antibiotics more often than expected at discharge and used longer durations than expected when antibiotics were prescribed. Patients at these group 4 hospitals were also less likely to receive the shortest effective duration for CAP relative to patients at other sites. In all, these findings suggest that some hospitals were more successful than others at reducing antibiotic overuse at hospital discharge.

Our work is novel in that we predicted antibiotic use at hospital discharge using encounter-level data. Several research teams have used encounter-level data to model inpatient antibiotic exposure, finding that this patient-level information is more predictive than facility- or unit-level variables [12, 16, 18]. Our study complements this prior work. Overall, it appears that patient characteristics, such as comorbid conditions and diagnoses of infection, help predict the risk of antibiotic exposure, whether it be prescribed as an inpatient or at discharge.

To our knowledge, we are the first to describe risk-adjustment methods for benchmarking hospitals on their overall antibiotic use at discharge. By adjusting for differences in case mix, our model may be “leveling the playing field” and, in turn, permitting interhospital comparisons of true discretionary practice variation. Metrics that account for differences in case mix across hospitals have more buy-in from clinicians, which has important implications for implementation [19].

Prior work has shown that, in the absence of a specific intervention, post-discharge antibiotic duration is frequently excessive [6, 8, 20, 21]. In fact, even at hospitals in our study that had shorter-than-expected post-discharge LOT (groups 1 and 2), antibiotic duration was still probably excessive in more than half of patients with CAP. This suggests that antibiotic overuse at discharge is widespread and that there is room for improvement, even at higher-performing sites.

By providing an assessment of how hospitals compare to their peers on overall post-discharge antibiotic use, our metric could motivate hospitals to perform a more robust assessment of antibiotic prescribing at discharge and to implement improvement processes. Improved antibiotic prescribing at discharge can, in turn, improve patient safety. In 1 study of 6481 patients with pneumonia across 43 Michigan hospitals, each excessive day of antibiotic therapy at discharge increased the risk of an antibiotic-related adverse event by 5% [22]. Prior to implementation of a pharmacist-driven practice model across 5 hospitals, 9% of patients who received a post-discharge antibiotic developed a severe antibiotic-related adverse event [20]. Efforts to improve the selection, dosing, and duration of post-discharge antibiotics can reduce the frequency of these adverse events [20].

While our risk-adjusted metric could have real-world applications, it is important to recognize its shortcomings. For one, we used 4 years of data to develop this method, but the metric would need to be calculated more frequently to provide meaningful feedback. We also acknowledge that the risk-adjusted metric does not tell a hospital how antibiotics should be prescribed but instead shows how a hospital's post-discharge antibiotic use compares to other facilities. Finally, the risk-adjusted metric does not identify a particular type of infection for which antibiotics are being excessively used. Syndrome-specific metrics, which capture far fewer patients, can be more actionable and could be complementary to our risk-adjusted metric. Our metric could also accompany other metrics, like the Standardized Antimicrobial Administration Ratio, that compare hospitals on their overall inpatient antibiotic use [23].

Due to difficulties with collecting certain data elements, our risk-adjustment metric may not be immediately generalizable to non-VHA settings, but there could be potential to apply similar approaches in the future. For example, the NHSN's implementation of Fast Healthcare Interoperability Resources (FHIR; Health Level Seven International, Ann Arbor, MI) will provide the potential for patient-level risk adjustment in its different modules, including the Antimicrobial Use and Resistance module. While our data reflect which antibiotics were actually dispensed to the patient, most non-VHA hospitals would only have access to electronic prescribing data sent to community pharmacies. Given the above-mentioned differences between VHA and non-VHA settings, external validation of our methods would be a necessary next step.

There are several limitations of our study that need to be acknowledged. First, our model excluded patients who received 30 days or more of an antibiotic at discharge. These patients accounted for only 7.7% of all patients who received post-discharge antibiotics, so the effect of exclusion was likely minimal. As outlined in the Methods section, we believe that the exclusion of these patients was important to ensure the “fairness” of the model. Second, we were unable to capture post-discharge parenteral antibiotics because these antibiotics are typically not dispensed by the VHA, even when they are recommended by VHA inpatient providers. Because outpatient parenteral antibiotics are infrequently recommended, the overall effect of these missing data is likely small. Third, our model only predicted post-discharge antibacterial exposure, not antiviral or antifungal exposure. Fourth, our measurement of uncomplicated CAP did not account for situations when certain bacterial organisms (eg, methicillin-resistant *Staphylococcus aureus*, *Pseudomonas aeruginosa*) were isolated from a respiratory culture that would justify a longer course of treatment [17]. Prior work in the VHA has shown that these 2 bacteria are isolated infrequently in hospitalized patients with CAP [24]. Therefore, the absence of these data was unlikely to have substantially biased our results.

In conclusion, we have developed a model using electronically available data that was able to predict antibiotic use prescribed at hospital discharge. Further development and refinement of similar models may provide a mechanism for giving feedback to hospitals on antibiotic overuse at this transition of care.

## Supplementary Data

Supplementary materials are available at *Clinical Infectious Diseases* online. Consisting of data provided by the authors to benefit the reader, the posted materials are not copyedited and are the sole responsibility of the authors, so questions or comments should be addressed to the corresponding author.

## Supplementary Material

ciae224_Supplementary_Data

## Notes

***Author Contributions.*** D. J. L., M. B. G., and M. G. conceived and designed the study. D. J. L., J. A. M., H. C., B. A., and B. B. contributed to the acquisition, analysis, or interpretation of data: D. J. L. drafted the manuscript. All authors critically revised the manuscript and gave final approval.

***Data availability.*** Data not publicly available.

***Financial support.*** This work was supported by an investigator-initiated research (IIR) grant from the VA Health Services Research and Development Service (IIR 20-280; Principal Investigator: D. J. L.).
