# Supplementary material for: A Novel Risk-Adjusted Metric to Compare Hospitals on Their Antibiotic Prescribing at Hospital Discharge
Source: Clin Infect Dis. 2024 Apr 24;79(3):588–95. doi: 10.1093/cid/ciae224 (PMC11426263; doi:10.1093/cid/ciae224)
Supplement: ciae224_Supplementary_Data [file ciae224_supplementary_data.docx]

**Table of Contents for Supplemental Material**

| **Supplemental item** | **Page number** |
| --- | --- |
| Supplemental Table 1. Description of how certain variables in the predictive model were collected | 2 |
| Supplemental Table 2a. Coefficient summary from the count model for post-discharge antibiotic use, 2018-2019 versus 2020-2021 | 3 |
| Supplemental Table 2b. Coefficient summary from the zero-inflated model for post-discharge antibiotic use, 2018-2019 versus 2020-2021 | 4 |
| Supplemental Table 3. Inclusion and exclusion criteria used to define cases of uncomplicated community-acquired pneumonia | 5 |
| Supplemental Table 4. International Classification of Diseases, Tenth Revision (ICD-10) diagnostic codes used to define cases of uncomplicated community-acquired pneumonia | 6 |
| Supplemental Table 5. Comparison between acute-care patients who were prescribed ≥ 30 of post-discharge antibiotic duration versus 1-29 days | 7-8 |
| Supplemental Table 6. Coefficient summary from the count and zero-inflated models for post-discharge antibiotic use | 9 |
| Supplemental Figure 1. Concordance between observed and predicted post-discharge length of antibiotic therapy | 10 |

**Supplemental Table 1. Description of how certain variables in the predictive model were collected**

| **Variable type** | **Description** |
| --- | --- |
| **Body mass index (BMI)** | BMI was calculated using the following data elements: height was collected with no specific look-back period and weight was collected closest to the patient’s admission. Based on the CDC’s formula, BMI = (height in pounds / (weight in inches)^2^)*703. |
| **Comorbidities** | ICD-10-CM inpatient and outpatient diagnostic codes from the prior 12 months and from the index admission were collected based on a modified version of the Elixhauser comorbidity index.^1^ |
| **Immunosuppressive medications** | - Receipt of chemotherapeutic agents within 30 days before the admission or during the admission; - Receipt of a variety of immune-modulating medications within 90 days before the admission or during the admission itself (e.g. azathioprine, calcineurin inhibitors, TNF-alpha inhibitors, corticosteroids, tyrosine kinase inhibitors, anti-CD52 agents, interleukin-1 receptor antagonist, immune checkpoint inhibitors, mTOR kinase inhibitors, mycophenolate mofetil, etc.). |
| **Immunosuppressive conditions** | The presence of an inpatient or outpatient diagnosis code for an immunosuppressive condition within the 12 months before admission or during the admission. These codes included diagnoses of leukemia, lymphoma, human immunodeficiency virus infection, bone marrow transplantation or solid organ transplantation. |

Reference:

1. Quan  H, Sundararajan  V, Halfon  P,  et al.  Coding algorithms for defining comorbidities in *ICD-9-CM* and *ICD-10* administrative data. *Med Care*. 2005;43(11):1130-1139.

**Supplemental Table 2a. Coefficient summary from the count model for post-discharge antibiotic use, 2018-2019 versus 2020-2021**

## Count model - Count model summary - LOT early (2018-19) versus late (2020-2021)

(Coefficients with discrepant significance levels are marked with an asterisk)

| **Variable** | **Coefficient (2018-19)** | **p-value** | **Coefficient**  **(2020-21)** | **p-value** |
| --- | --- | --- | --- | --- |
| (Intercept) | 1.872 | <0.001 | 1.884 | <0.001 |
| **Age** | -0.004 | <0.001 | -0.004 | <0.001 |
| **Male sex** | 0.061 | <0.001 | 0.048 | <0.001 |
| **Body Mass Index** |  |  |  |  |
| Missing | 0.017 | 0.286 | -0.020 | 0.277 |
| Normal | 0.013 | 0.360 | -0.012 | 0.467 |
| Overweight/obese | 0.013 | 0.346 | -0.022 | 0.205 |
| **Transferred in from another hospital** | 0.013 | 0.356 | 0.028 | 0.105 |
| **Medical specialty at discharge** | 0.150 | <0.001 | 0.149 | <0.001 |
| **Immunosuppressive medications** | 0.056 | <0.001 | 0.064 | <0.001 |
| **Comorbidities** |  |  |  |  |
| Congestive heart failure | -0.023 | <0.001 | -0.017 | 0.006 |
| Chronic lung disease* | -0.013 | 0.010 | 0.005 | 0.332 |
| Diabetes mellitus | 0.011 | 0.014 | 0.022 | <0.001 |
| Immunodeficiency | 0.079 | <0.001 | 0.047 | <0.001 |
| Liver disease | -0.002 | 0.768 | -0.008 | 0.235 |
| Metastatic cancer* | 0.021 | 0.023 | 0.012 | 0.223 |
| Neurological disorder | -0.046 | <0.001 | -0.037 | <0.001 |
| Peptic ulcer disease | 0.050 | <0.001 | 0.073 | <0.001 |
| Pulmonary circulation disorders | 0.012 | 0.252 | 0.012 | 0.278 |
| Peripheral vascular disease | 0.030 | <0.001 | 0.032 | <0.001 |
| Renal failure or on dialysis | -0.002 | 0.751 | -0.008 | 0.159 |
| Rheumatic disorder* | 0.003 | 0.805 | 0.028 | 0.046 |
| Substance abuse | -0.024 | <0.001 | -0.006 | 0.310 |
| Valvular heart disease | 0.000 | 0.945 | 0.002 | 0.808 |
| Weight loss | 0.032 | <0.001 | 0.030 | <0.001 |
| **Discharge diagnoses** |  |  |  |  |
| COPD, acute exacerbation | -0.299 | <0.001 | -0.299 | <0.001 |
| Miscellaneous bacterial infections | 0.218 | <0.001 | 0.221 | <0.001 |
| Pneumonia | -0.117 | <0.001 | -0.154 | <0.001 |
| Skin and soft tissue infections | 0.092 | <0.001 | 0.085 | <0.001 |
| Urinary tract infections* | 0.044 | <0.001 | 0.012 | 0.074 |
| Intra-abdominal infection | 0.157 | <0.001 | 0.131 | <0.001 |
| Osteoarticular infections | 0.258 | <0.001 | 0.284 | <0.001 |
| **Inpatient antibiotic exposure** |  |  |  |  |
| 1-2 days* | -0.043 | <0.001 | -0.002 | 0.855 |
| 3-7 days* | -0.004 | 0.627 | 0.025 | 0.019 |
| > 7 days | 0.232 | <0.001 | 0.279 | <0.001 |
| No inpatient antibiotic exposure | -- | -- | -- | -- |

**Supplemental Table 2b. Coefficient summary from the zero-inflated model for post-discharge antibiotic use, 2018-2019 versus 2020-2021**

## Zero-inflated model - LOT early (2018-19) versus late (2020-2021)

(Coefficients with discrepant significance levels are marked with an asterisk)

| **Variable** | **Coefficient**  **(2018-19)** | **p-value** | **Coefficient**  **(2020-21)** | **p-value** |
| --- | --- | --- | --- | --- |
| (Intercept) | 2.587 | <0.001 | 2.988 | <0.001 |
| **Age** | 0.005 | <0.001 | 0.003 | <0.001 |
| **Male sex** | -0.049 | 0.018 | -0.111 | <0.001 |
| **Body Mass Index** |  |  |  |  |
| Missing | 0.205 | <0.001 | 0.214 | <0.001 |
| Normal | -0.015 | 0.649 | -0.018 | 0.627 |
| Overweight/obese | -0.045 | 0.174 | 0.008 | 0.829 |
| **Transferred in from another hospital** | 0.343 | <0.001 | 0.430 | <0.001 |
| **Medical specialty at discharge** | 0.637 | <0.001 | 0.468 | <0.001 |
| **Immunosuppressive medications*** | 0.031 | 0.172 | 0.098 | <0.001 |
| **Comorbidities** |  |  |  |  |
| Congestive heart failure | 0.231 | <0.001 | 0.171 | <0.001 |
| Chronic lung disease | -0.072 | <0.001 | -0.052 | <0.001 |
| Diabetes mellitus* | 0.027 | 0.043 | -0.005 | 0.739 |
| Immunodeficiency | 0.030 | 0.197 | -0.014 | 0.574 |
| Liver disease | 0.067 | <0.001 | 0.074 | <0.001 |
| Metastatic cancer | 0.065 | 0.044 | -0.093 | 0.006 |
| Neurological disorder | 0.504 | <0.001 | 0.517 | <0.001 |
| Peptic ulcer disease | 0.180 | <0.001 | 0.182 | <0.001 |
| Pulmonary circulation disorders | 0.287 | <0.001 | 0.216 | <0.001 |
| Peripheral vascular disease | 0.122 | <0.001 | 0.113 | <0.001 |
| Renal failure or on dialysis | 0.134 | <0.001 | 0.101 | <0.001 |
| Rheumatic disorder | 0.003 | 0.913 | 0.021 | 0.506 |
| Substance abuse | 0.125 | <0.001 | 0.079 | <0.001 |
| Valvular heart disease | 0.209 | <0.001 | 0.195 | <0.001 |
| Weight loss | 0.325 | <0.001 | 0.371 | <0.001 |
| **Discharge diagnoses** |  |  |  |  |
| COPD, acute exacerbation | -0.702 | <0.001 | -0.631 | <0.001 |
| Miscellaneous bacterial infections | -0.373 | <0.001 | -0.410 | <0.001 |
| Pneumonia | -0.886 | <0.001 | -0.655 | <0.001 |
| Skin and soft tissue infections | -0.804 | <0.001 | -0.838 | <0.001 |
| Urinary tract infections | -0.743 | <0.001 | -0.775 | <0.001 |
| Intra-abdominal infection | -0.823 | <0.001 | -0.905 | <0.001 |
| **Inpatient antibiotic exposure** |  |  |  |  |
| 1-2 days | -3.180 | <0.001 | -3.181 | <0.001 |
| 3-7 days | -3.404 | <0.001 | -3.397 | <0.001 |
| > 7 days | -1.902 | <0.001 | -1.917 | <0.001 |

**Supplemental Table 3. Inclusion and exclusion criteria used to define cases of uncomplicated community-acquired pneumonia**

|  |
| --- |
| **Inclusion criteria** |
| - Diagnosis of pneumonia coded at discharge^1^ - At least 4 unique calendar days of antibiotic exposure (inpatient +/- post-discharge) - Chest imaging done ≤ 24 hours prior to admission and ≤ 48 hours after admission ^2^ - Antibiotics initiated < 48 hours after admission |
|  |
|  |
|  |
| **Exclusion criteria** |
| - Transferred in from an outside hospital - Discharged to a nursing home, hospital or to hospice - Dead at discharge - Left against medical advice - Length of antibiotic therapy prescribed at discharge ≥ 30 days - Immunosuppressive medications^3^ - Immunosuppressive conditions^4^ - Pneumonia was diagnosed during the 30 days prior to admission - Any other bacterial infection coded at discharge^5^ - Positive culture from sterile body sites^6^ - Pleural drainage procedure up to 30 days prior to admission or during the admission - Complicated pneumonia, e.g., lung abscess, empyema - Positive Legionella urine antigen |
|  |
|  |
|  |
|  |
|  |
|  |
|  |
|  |
|  |
|  |
|  |
|  |
|  |

1. There were 28 ICD-10 codes used to define pneumonia (Supplemental Table 4).
2. For chest imaging, either a chest radiograph or a computed tomography scan of the chest qualified.
3. Data on the use of immunosuppressive medications was collected, including receipt of chemotherapeutic agents within 30 days before or during the admission and receipt of anti-rejection medications within 90 days before the admission or during the admission itself.
4. Immunosuppressive conditions were assessed by looking for the presence of an inpatient or outpatient diagnosis code for an immunosuppressive condition within the 12 months before admission or during the admission itself. These codes included diagnoses of leukemia, lymphoma, human immunodeficiency virus infection, bone marrow transplantation or solid organ transplantation.
5. Bacterial infections were defined by the Agency for Healthcare Research and Quality’s Patient Safety Indicators Appendix F, version 2022: Infection Diagnosis Codes.
6. Microbiologic data was available from the following sterile body sites: blood, cerebrospinal fluid, pleural fluid, and synovial fluid.

| **ICD-10 codes** | **Description** |
| --- | --- |
| J13 | Pneumonia due to Streptococcus pneumoniae |
| J14 | Pneumonia due to Hemophilus influenzae |
| J15 | Bacterial pneumonia, not elsewhere classified |
| J150 | Pneumonia due to Klebsiella pneumoniae |
| J151 | Pneumonia due to Pseudomonas |
| J152 | Pneumonia due to staphylococcus |
| J1520 | Pneumonia due to staphylococcus, unspecified |
| J1521 | Pneumonia due to staphylococcus aureus |
| J15211 | Pneumonia due to methicillin susceptible Staphylococcus aureus |
| J15212 | Pneumonia due to Methicillin resistant Staphylococcus aureus |
| J1529 | Pneumonia due to other staphylococcus |
| J153 | Pneumonia due to streptococcus, group B |
| J154 | Pneumonia due to other streptococci |
| J155 | Pneumonia due to Escherichia coli |
| J156 | Pneumonia due to other Gram-negative bacteria |
| J157 | Pneumonia due to Mycoplasma pneumoniae |
| J158 | Pneumonia due to other specified bacteria |
| J159 | Unspecified bacterial pneumonia |
| J16 | Pneumonia due to other infectious organisms, NEC |
| J160 | Chlamydial pneumonia |
| J168 | Pneumonia due to other specified infectious organisms |
| J17 | Pneumonia in diseases classified elsewhere |
| J18 | Pneumonia, unspecified organism |
| J180 | Bronchopneumonia, unspecified organism |
| J181 | Lobar pneumonia, unspecified organism |
| J188 | Other pneumonia, unspecified organism |
| J189 | Pneumonia, unspecified organism |
| A481 | Legionnaires' disease |

**Supplemental Table 4. International Classification of Diseases, Tenth Revision (ICD-10) diagnostic codes used to define cases of uncomplicated community-acquired pneumonia**

**Supplemental Table 5. Comparison between acute-care patients who were prescribed ≥ 30 of post-discharge antibiotic duration versus 1-29 days**

|  | **Post-discharge antibiotics**  **LOT ≥ 30 days**  **(n=27,413)** | **Post-discharge antibiotics**  **LOT 1-29 days**  **(n=323,943)** |
| --- | --- | --- |
| Age, mean (SD) | 66.2 (11.6) | 68.5 (12.7) |
| Male sex, n (%) | 26,060 (95.1) | 304,517 (94.0) |
| Body mass index  Normal  Overweight/obese  Missing  Underweight | 7,305 (26.7)  17,080 (62.3)  2,212 (8.1)  816 (3.0) | 76,494 (23.6)  216,309 (66.8)  22,230 (7.2)  7,910 (2.4) |
| Transferred in from another hospital | 975 (3.6) | 6,578 (2.1) |
| Medical specialty at discharge | 22,022 (80.3) | 263,971 (81.5) |
| Comorbidities^1^  Congestive heart failure  Chronic lung disease  Diabetes mellitus  Immunodeficiency^2^  Liver disease  Metastatic cancer  Neurological disorder^3^  Peptic ulcer disease  Pulmonary circulation disorders  Peripheral vascular disease  Renal disease or dialysis  Rheumatic disorder  Substance abuse^4^  Valvular disease  Weight loss | 7,942 (29.0)  11,261 (41.1)  13,423 (49.0)  5,030 (18.4)  7,079 (25.8)  2,093 (7.6)  5,773 (21.1)  1,314 (4.8)  1,698 (6.2)  7,784 (28.4)  8,801 (32.1)  1,155 (4.2)  8,195 (29.9)  3,308 (12.1)  6,289 (22.9) | 91,460 (28.2)  143,248 (44.2)  147,936 (45.7)  17,592 (5.4)  51,367 (15.9)  19,529 (6.0)  61,772 (19.1)  9,714 (3.0)  15,366 (4.7)  74,670 (23.1)  86,483 (26.7)  9,954 (3.1)  73,794 (22.8)  34,599 (10.7)  43,824 (13.4) |
| Immunosuppressive medications^5^ | 4,649 (17.0) | 17,402 (5.4) |
| Discharge diagnoses  COPD exacerbation  Intra-abdominal and biliary infections  Miscellaneous bacterial infections^6^  Osteoarticular infection  Pneumonia  Skin & soft tissue infection  Urinary tract infection | 2,739 (10.0)  2,635 (9.6)  13,155 (48.0)  5,666 (20.7)  3,398 (12.4)  6,041 (22.0)  3,143 (11.5) | 50,274 (15.5)  27,459 (8.5)  119,761 (37.0)  12,626 (3.9)  65,620 (20.3)  65,686 (20.3)  60,619 (18.7) |
| Inpatient antibiotic exposure  None  1-2 days  3-7 days  >7 days | 1,823 (6.7)  4,408 (16.1)  12,755 (46.5)  8,427 (30.7) | 20,987 (6.5)  112,440 (34.7)  167,449 (51.7)  23,067 (7.1) |

LOT = length of therapy

1. These comorbidities were selected because a prior Delphi panel determined that they were causally or indeterminately related to appropriate inpatient antibiotic use.^12^ Note that some conditions identified by the Delphi panel have been combined together.
2. Immunodeficiency includes diagnoses of leukemia, lymphoma, human immunodeficiency virus infection, bone marrow transplantation or solid organ transplantation.
3. Neurological disorders include paralysis and other neurological disorders, e.g. Parkinson’s disease, multiple sclerosis, epilepsy, etc.
4. Substance abuse includes alcohol and/or drug abuse.
5. Immunosuppressive medications included receipt of chemotherapeutic agents within 30 days before the admission or during the admission itself or receipt of anti-rejection medications within 90 days before the admission or during the admission itself.
6. Miscellaneous infections included endocarditis, central nervous system infections, complicated pneumonia (e.g. empyema, lung abscess), sepsis and any other bacterial infections that were not otherwise categorized.

**Supplemental Table 6. Coefficient summary from the count and zero-inflated models for post-discharge antibiotic use**

|  | **Count model** | | **Zero-inflated model** | |
| --- | --- | --- | --- | --- |
| **Variable** | **Coefficient** | **p-value** | **Coefficient** | **p-value** |
| **Patient age** | -0.004 | <0.001 | -0.002 | <0.001 |
| **Male sex** | 0.056 | <0.001 | 0.054 | <0.001 |
| **Body Mass Index**  Normal  Overweight/obese  Missing  Underweight (reference) | 0.003  -0.002  -0.001  --- | 0.807  0.882  0.912  --- | 0.017  0.019  -0.191  --- | 0.475  0.442  <0.001  --- |
| **Transferred in from another hospital** | 0.020 | 0.079 | -0.401 | <0.001 |
| **Surgical specialty at discharge** | 0.151 | <0.001 | -0.561 | <0.001 |
| **Immunosuppressive medications** | 0.059 | <0.001 | -0.053 | <0.001 |
| **Comorbidities**  Congestive heart failure  Chronic lung disease  Diabetes mellitus  Immunodeficiency  Liver disease  Metastatic cancer  Neurological disorders  Peptic ulcer disease  Peripheral vascular disease  Pulmonary circulation disorders  Renal failure or on dialysis  Rheumatic disorder  Substance abuse  Valvular heart disease  Weight loss | -0.021  -0.004  0.016  0.058  -0.005  0.016  -0.042  0.060  0.031  0.012  -0.004  0.014  -0.017  0.001  0.031 | <0.001  0.242  <0.001  <0.001  0.250  0.017  <0.001  <0.001  <0.001  0.119  0.321  0.139  <0.001  0.854  <0.001 | -0.224  0.031  -0.009  -0.01  -0.083  -0.099  -0.632  -0.171  -0.071  -0.279  -0.133  -0.036  -0.107  -0.213  -0.325 | <0.001  <0.001  0.224  0.169  <0.001  <0.001  <0.001  <0.001  <0.001  <0.001  <0.001  0.080  <0.001  <0.001  <0.001 |
| **Discharge diagnoses**  COPD, acute exacerbation  Intra-abdominal and biliary infection  Miscellaneous bacterial infections  Osteoarticular infections  Pneumonia  Skin and soft tissue infection  Urinary tract infection | -0.298  0.144  0.220  0.271  -0.132  0.089  0.029 | <0.001  <0.001  <0.001  <0.001  <0.001  <0.001  <0.001 | 0.673  0.859  0.459  -0.64  0.721  0.889  0.684 | <0.001  <0.001  <0.001  <0.001  <0.001  <0.001  <0.001 |
| **Inpatient antibiotic exposure**  1-2 days  3-7 days  > 7 days  No inpatient antibiotic exposure | -0.025  0.008  0.251  --- | <0.001  0.263  <0.001  --- | 3.193  3.45  2.019  --- | <0.001  <0.001  <0.001  --- |

**Supplemental Figure 1. Concordance between observed and predicted post-discharge length of antibiotic therapy**


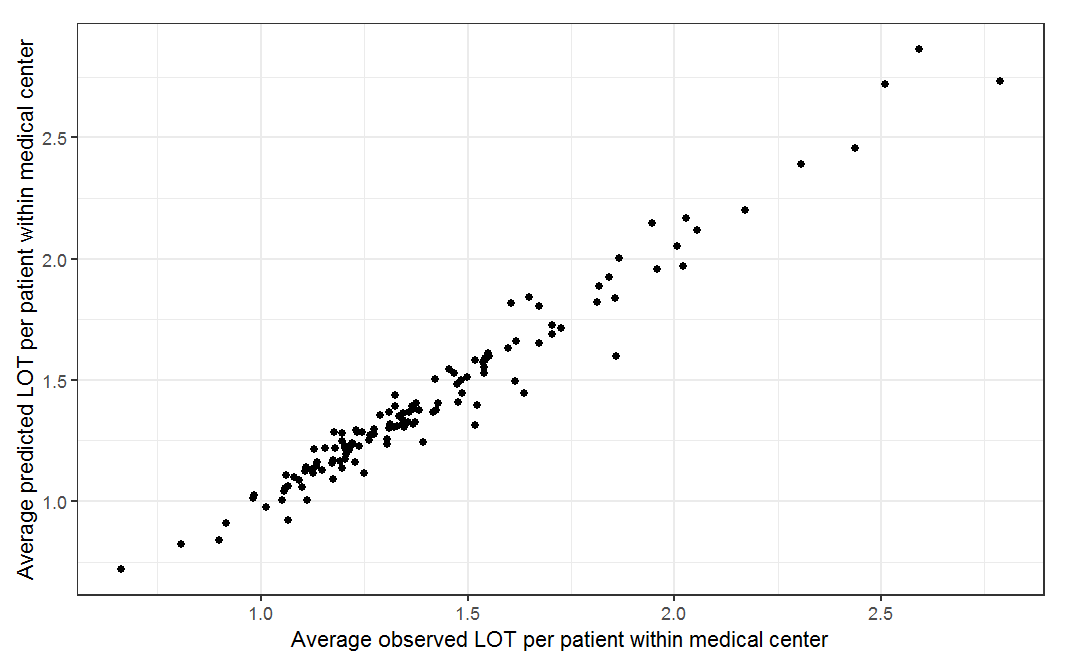


Each dot represents the average of either the observed LOT or the LOT predicted by the model within a single medical center.
